# The Role of Uridine Adenosine Tetraphosphate in the Vascular System

**DOI:** 10.1155/2011/435132

**Published:** 2011-11-01

**Authors:** Takayuki Matsumoto, Rita C. Tostes, R. Clinton Webb

**Affiliations:** ^1^Department of Physiology, Georgia Health Sciences University, 1120 Fifteenth Street, CA-3135, Augusta, GA 30912-3000, USA; ^2^Department of Physiology and Morphology, Institute of Medicinal Chemistry, Hoshi University, 2-4-41 Ebara, Shinagawa-ku, Tokyo 142-8501, Japan; ^3^Department of Pharmacology, School of Medicine of Ribeirao Preto, University of Sao Paulo, 14049-900 Sao Paulo, SP, Brazil

## Abstract

The endothelium plays a pivotal role in vascular homeostasis, and endothelial dysfunction is a major feature of cardiovascular diseases, such as arterial hypertension, atherosclerosis, and diabetes. Recently, uridine adenosine tetraphosphate (Up_4_A) has been identified as a novel and potent endothelium-derived contracting factor (EDCF). Up_4_A structurally contains both purine and pyrimidine moieties, which activate purinergic receptors. There is an accumulating body of evidence to show that Up_4_A modulates vascular function by actions on endothelial and smooth muscle cells. In this paper, we discuss the effects of Up_4_A on vascular function and a potential role for Up_4_A in cardiovascular diseases.

## 1. Introduction 

A healthy endothelium expresses and releases various molecules, which aid to maintain vascular structure and homeostasis [1, 2]. Endothelial cells actively regulate basal vascular tone and vascular reactivity in physiological and pathophysiological conditions, by responding to mechanical forces (e.g., shear stress) and neurohumoral mediators with the release of a variety of relaxing factors [endothelium-derived relaxing factors (EDRFs)] or contracting factors [endothelium-derived contracting factors (EDCFs)] [3–5]. Endothelial dysfunction plays a key role in the initiation and development of both macro- and microangiopathy in patients with arterial hypertension, inflammatory-associated diseases (atherosclerosis), hypercholesterolemia, stroke, diabetes, as well as in animal models of these diseases [4–12]. The mechanisms that regulate the balance between EDRFs and EDCFs are important for vascular health. Mechanisms that increase EDRFs and/or decrease the release and/or bioavailability of EDCFs are promising drug targets to alleviate the damage caused by endothelial dysfunction. So far, several factors are known as EDCFs such as endothelin-1, angiotensin II, vasoconstrictor prostanoids, and reactive oxygen species [4, 5, 13]. 

**Figure 1:**
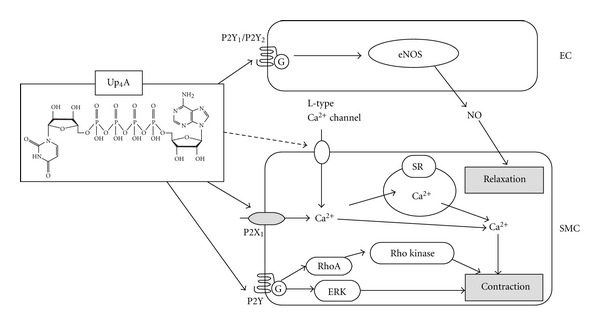
Up_4_A and vascular tone. Details are shown in the text. Up_4_A may directly or indirectly activate L-type Ca^2+^ channel. NO: nitric oxide; eNOS: endothelial nitric oxide synthase; EC: endothelial cell; SMC: smooth muscle cell; SR: sarcoplasmic reticulum; ERK: extracellular signal regulated kinase.

The dinucleotide uridine adenosine tetraphosphate (Up_4_A) (Figure 1) was identified by Jankowski et al. [14] as a novel potent EDCF. Up_4_A was isolated from the supernatant of stimulated human endothelium and was identified by mass spectrometry. Up_4_A is released from the endothelium in response to acetylcholine (ACh), the calcium ionophore (A23187), endothelin-1, adenosine triphosphate (ATP), uridine triphosphate (UTP), and mechanical stress. Therefore, Up_4_A can contribute to vascular regulation as an endothelium-derived factor [14]. Up_4_A plasma concentrations detected in healthy subjects are high enough to cause vasoconstriction [14]. Moreover, Up_4_A is the first dinucleotide found in living organisms that contains both purine and pyrimidine moieties (Figure 1).

 Purinergic signaling is important not only in short-term regulation of vascular tone but also in long-term regulation of vascular remodeling (i.e., vascular cell proliferation, migration, and death) [15–21]. Moreover, dinucleotides containing two purine moieties are known, and their role in vasomotor regulation is increasingly recognized [20, 22, 23]. However, the vasoactivity of Up_4_A may differ from those of dinucleotides exclusively containing purines [20, 24]. Therefore, Up_4_A can play a functional role in the vascular system both under physiological and pathophysiological conditions.

This paper focuses on the effects of Up_4_A on vascular tone and its putative role on vascular function.

## 2. Up_4_A and Vascular Tone under Physiological Conditions

Several reports demonstrated that Up_4_A modulates vasomotor activity in vessels from nondisease animal models using both *in vitro* (i.e., perfusion or myograph system) and *in vivo* techniques (Table 1, Figure 1). Since Up_4_A possesses both purine and pyrimidine moiety, these studies mainly focus on the relationship between Up_4_A and purinoceptor signaling. 

Purinoceptors have been classified into two subtypes (namely, P1 receptors (or adenosine receptors) and P2 receptors) based on their molecular cloning and pharmacological properties [25–28]. Adenosine and its phosphates, ATP and ADP, have been identified as the endogenous ligand for P1 and P2 receptors, respectively. Four subtypes of metabotropic P1 receptors have been cloned and named A1, A2A, A2B, and A3 [25, 27]. The P2 receptors exist in two major families: ionotropic (P2X receptors) and metabotropic (P2Y receptors) [26–30]. Currently, there are at least 7 cloned P2X receptors and at least 8 cloned P2Y receptors [15–18, 21, 25–30]. P2X receptors are mainly activated by ATP and its analogs, whereas P2Y receptors can be activated by ATP, UTP, and UDP, depending on the subtypes of P2Y receptors involved [15–18, 21, 25–30]. Most of these receptors are capable of mediating responses to several nucleotides, resulting in multiple receptors having overlapping ligand preferences. In addition, distribution of these receptors varies among different tissues [15–18, 21, 25–30]. Therefore, purinergic signaling is complicated. In this section, we will describe reports suggesting that Up_4_A affects vascular tone and discuss the relevant mechanisms involved in Up_4_A responses. 

### 2.1. Aorta (Rat)

Linder et al. [31] characterized the effect of Up_4_A in thoracic aorta from rats using isometric tension recording. In intact aortic rings precontracted with phenylephrine, Up_4_A treatment led to a modest endothelium-dependent relaxation. On the other hand, under basal conditions, Up_4_A induced a concentration-dependent contraction. This contraction was potentiated by endothelium denudation or nitric oxide synthase (NOS) inhibition suggesting that EDRF (mainly NO) constitutively suppresses Up_4_A-induced contraction in thoracic aorta. Linder et al. [31] further found that Up_4_A-induced contraction was suppressed by P1 (8-PST [32]) or P2X (NF279 [33]) receptor antagonists, L-type Ca^2+^ channel blockade (nifedipine [34]), and Rho-kinase inhibition (Y27632 [35]). These results suggested that Up_4_A-induced contraction is modulated by NO, mediated by P1 and P2X receptor activation, and involves L-type Ca^2+^ channels and Rho-kinase activation in smooth muscle cells. Moreover, Up_4_A-induced contraction was attenuated by a membrane permeable superoxide scavenger (tempol) and by an NADPH oxidase inhibitor (apocynin) suggesting that superoxide generation affects Up_4_A-induced contractile responses [31].

### 2.2. Pulmonary Artery (Rat)

Gui et al. [36] characterized the effect of Up_4_A in pulmonary artery from rats using isometric tension recording and investigated the signaling mechanisms related to Up_4_A responses. Up_4_A induced concentration-dependent contraction of isolated rat pulmonary arteries. Up_4_A was as potent as UTP and UDP in endothelium-denuded arteries, while much more effective than UTP and UDP in endothelium-intact preparations [36]. Up_4_A -induced contraction was blocked by suramin, but not by P2X receptor antagonist (Ip_5_I) or desensitization of P2X receptors with *α*,*β*-methylene-ATP [36]. Up_4_A -induced contraction was inhibited by pretreatment with an inhibitor of Ca^2+^ release from sarcoplasmic reticulum (thapsigargin), a Ca^2+^ channel blocker (nitrendipine) and a Ca^2+^ chelator (EGTA), but unaffected by a Rho-kinase inhibitor (H-1152) [36]. Moreover, unlike ATP and UTP, Up_4_A does not induce vasodilation of endothelium-intact preparations contracted with phenylephrine [36]. These results suggest that Up_4_A is a potent vasoconstrictor, but not a vasodilator in the rat pulmonary artery, and such contraction is mainly via a suramin-sensitive P2Y receptor. The contractile effect of Up_4_A involves the entry of extracellular Ca^2+^ and release of Ca^2+^ from intracellular stores but not Ca^2+^ sensitization due to the activation of RhoA/Rho kinase pathway in vascular smooth muscle cells. Therefore, Up_4_A potentially plays an important role in the regulation of pulmonary vascular tone.

### 2.3. Renal Artery (Rat and Mouse)

Jankowski et al. [14] observed that in rat isolated perfused kidney, Up_4_A stimulated vasoconstriction mainly via P2X_1_ receptors and probably also via P2Y_2_ and P2Y_4_ receptors. Very recently, findings from this same group indicate that in the rat perfused kidney, in addition to smooth muscle P2X_1_ receptor-mediated vasoconstriction, Up_4_A showed concentration-dependent P2Y_2_ receptor-mediated, long-lasting vasoconstriction [37]. Moreover, they demonstrated that Up_4_A-induced vasoconstriction was followed by vasodilation mediated by P2Y_1_ and P2Y_2_ receptor activation on endothelial cells leading to the release of NO [37].

In mouse vessels, Up_4_A acts as a strong vasoconstrictive mediator on afferent arterioles, but has no significant effect on the tone of efferent arterioles [38]. The selective preglomerular vasoconstrictor activity of Up_4_A may be due to the lack of P2X_1_ receptors, which are the main target of Up_4_A, in postglomerular arterioles [39]. Therefore, it may be assumed that Up_4_A contributes to the regulation of glomerular perfusion, intraglomerular pressure, and glomerular filtration rate. Moreover, Up_4_A was synthesized/secreted not only by the endothelium but also by renal tubular cells. Stimulation of tubule cells with oleoyl-2-acetyl-sn-glycerol (OAG, protein kinase C activator) increases the release rate of Up_4_A from tubule cells approximately 10-fold [38]. The release of Up_4_A from renal tubular cells may affect renal perfusion. Up_4_A release may further contribute to renal vascular autoregulation mechanisms [19, 40]. 

These results suggest that Up_4_A may play an important role in renal haemodynamics and blood pressure regulation.

### 2.4. Aorta (Mouse)

Hansen et al. [41] characterized the effects of Up_4_A in aorta from mice using isometric tension recording and *in vivo* arterial pressure measurements in conscious mice and rats (see below). Up_4_A has both relaxing and contracting effects depending on the Up_4_A concentration, the presence of precontraction, and the mode of stimulation (namely, single versus cumulative dose/concentrations). Up_4_A produced contraction in mouse aorta. In rings precontracted with phenylephrine, Up_4_A induced relaxation. A pronounced transient contraction was observed when 10^−5^ M Up_4_A was added as a bolus, while vasodilation was predominant when Up_4_A was added cumulatively. The contraction induced by low concentrations of Up_4_A was abolished by a cyclooxygenase inhibitor (indomethacin), suggesting that Up_4_A-induced contraction may be attributable to cyclooxygenase metabolites. Therefore, Up_4_A can evoke both relaxation and contraction in mouse aorta as well as rat aorta [31].

### 2.5. Up_4_A Affects Arterial Blood Pressure *In Vivo* (Rat and Mouse)

Jankowski et al. [14] investigated the effects of Up_4_A on mean arterial pressure of rats. Both noradrenaline and Up_4_A increased mean arterial pressure when injected intra-aortically in the anesthetized rat. Although noradrenaline evoked a sharp, short-lasting increase in arterial blood pressure, the same amount of Up_4_A showed a more prolonged effect on arterial blood pressure. 

Hansen et al. [41] determined the effects of Up_4_A on mean arterial pressure in conscious mice and rats. Intravenous infusion of increasing doses of Up_4_A to unrestrained mice and to conscious, trained rats caused a decrease in mean arterial pressure at higher rates of administration concomitant with a marked antinatriuretic effect. The discrepancy between the two studies may be explained by differences between the methodologies (namely, unconscious versus conscious) and by differences in the administration of Up_4_A (namely, single dose versus step-up doses). Future experiments need to be performed to address this question.

Table 1 and Figure 1 summarize Up_4_A effects on various arteries from different species.

## 3. Up_4_A and Pathophysiological States

There is evidence that Up_4_A might have implications in the pathogenesis of human arterial hypertension. Jankowski et al. [42] demonstrated that the plasma concentrations of Up_4_A are increased in juvenile hypertensive humans compared with normotensive subjects. Up_4_A concentration significantly correlates with the left ventricular mass and intima/media wall thickness in the hypertensive patients [42]. Therefore, Up_4_A may have an association with hypertension and hypertension-related vascular abnormalities.

As mentioned above, so far, the studies of Up_4_A-mediated responses have been carried out only in normal animals, and there is no study to indicate the vascular effects of Up_4_A under pathophysiological conditions, such as arterial hypertension. Since the vascular responsiveness to Up_4_A in hypertensive states remains unexplored/unknown, we [43] recently addressed this issue using deoxycorticosterone acetate-salt (DOCA-salt) rats, a well-known salt-dependent experimental model of arterial hypertension [44–47]. Using isometric tension recording (myograph), we observed that Up_4_A produced concentration-dependent contractions in segments of renal and pulmonary arteries at basal resting tension [43]. In DOCA-salt rats [versus its control uninephrectomized (Uni) rats], Up_4_A-induced contraction was similar in pulmonary artery and greater in renal artery [43]. Up_4_A-induced contraction in renal artery from both DOCA-salt and control groups was inhibited by a nonselective P2 receptor antagonist (suramin) but not by a P2X receptor antagonist (Ip_5_I). Furthermore, selective P2Y_2_ agonist-(2-Thio-UTP-), P2Y_2_/P2Y_4_ agonist-(UTP*γ*S-), and P2Y_6_ agonist-(MRS2693-) induced contractions were all increased in renal artery from DOCA-salt rats. Renal arterial protein expression of P2Y_2_, P2Y_4_, and P2Y_6_ receptors was similar between the two groups. The extracellular signal regulated kinase (ERK) pathway plays important roles in the regulation of vascular tone [46, 48–50], and it has been demonstrated that P2Y receptor activation can induce ERK pathway activation [18, 28, 51]. In DOCA-salt renal artery, the enhanced Up_4_A-induced contraction was reduced by an ERK pathway inhibitor (PD98059), and ERK activation stimulated by Up_4_A was enhanced in renal artery from DOCA-salt rats. Enhanced P2Y receptor signaling and activation of the ERK pathway represent likely mechanisms mediating the augmented Up_4_A-induced contraction in renal artery from DOCA-salt hypertensive rats. Moreover, we recently observed that, in DOCA-salt rats (versus Uni rats), Up_4_A-induced contraction was increased in basilar and femoral arteries, was decreased in small mesenteric artery, and was unchanged in thoracic aorta [52]. These results suggest that Up_4_A-induced contraction is heterogeneously affected among several vascular beds in DOCA-salt hypertensive rats. These results indicate that abnormal Up_4_A-induced contraction may be associated with the vascular dysfunction seen in hypertension. 

Jankowski et al. [42] also found that Up_4_A could lead to proliferation of human vascular smooth muscle cells (VSMCs). This cell-cycle-dependent process involves stimulation of S phase entry, and is due to the activation of P2Y receptor rather than P2X receptor. Very recently, Gui et al. [53] also suggested that Up_4_A stimulated proliferation of VSMCs via activation of P2Y receptors and the PI3-kinase/Akt and mitogen-activated protein kinase (MAPK) pathways. Since increased proliferation of VSMCs reflects not only on intima/media wall thickness but also on atherogenesis [54–57], Up_4_A may play a potential role in the development of atherosclerosis. 

Schuchardt et al. [58] investigated the influence of Up_4_A on formation of monocyte chemoattractant protein-1 (MCP-1), which is an important early component of the inflammatory response in atherosclerosis and induced by oxidative stress [56, 59–61]. The authors also characterized the underlying signaling transduction mechanisms in rat VSMCs. Up_4_A induced MCP-1 expression and secretion in VSMCs through the activation of P2Y_2_ receptor in a concentration-dependent manner. MCP-1 formation depended on generation of ROS. Up_4_A-induced MCP-1 formation was suppressed by NAD(P)H oxidase inhibitors (apocynin and diphenyl-iodonium) and by siRNA against NOX1 (a component of NAD(P)H oxidase [62–64]). Moreover, Up_4_A stimulated Rac1 activation and p47^phox^ translocation from cytosol to the plasma membrane (these processes are required for assembling and activation of NOX). The activation of MAPKs (i.e., ERK1/2 and p38 MAPK) is essential for Up_4_A-mediated intracellular signal transduction [58]. These results clearly demonstrated that Up_4_A induces NOX1-dependent ROS production, which further stimulates MCP-1 formation through MAPK phosphorylation in VSMCs. This process requires the activation of P2Y_2_ receptor. Thus, Up_4_A is not only a potent EDCF but also a potent inducer of proinflammatory response in the vascular wall. 

Moreover, Up_4_A has a stimulatory effect on the oxidative burst response (ROS production) of nonstimulated and *N*-formyl-methionine-leucine-phenylalanine-(fMLP-) activated monocytes as well as after phorbol 12-myristate 13-acetate (PMA) stimulation of both monocytes and lymphocytes [65]. Chronic inflammation in chronic kidney disease or atherosclerosis is associated with oxidative stress, and leukocytes are an important source of ROS [56, 57, 66]. Up_4_A potentially has impact on the initiation and progression of vascular inflammatory diseases and may represent a linking between blood pressure regulation and atherosclerosis.

## 4. Conclusions

The present work reviews reported studies on the effects of Up_4_A on vascular function in physiological and pathophysiological states. Although Up_4_A definitely has an important role in vascular function, some questions currently remain unresolved. For instance, what are the mechanisms of synthesis and catabolism of Up_4_A? To what extent are there regionally differences in Up_4_A kinetics? Are there mechanisms modified in vessels under physiological and pathophysiological states? How and to what extent do Up_4_A receptor(s) interact with Up_4_A putative degradation forms (e.g., mononucleotides and nucleotides) in the vascular system? How do the vascular actions of Up_4_A change during aging? Are there sex differences in the response to the dinucleotide? Since ion channels (e.g., P2X receptor) and G protein-coupled receptor (e.g., P2Y receptor and adenosine receptor) participate in multiprotein complexes with signaling molecules and other receptors (dimerized receptor or unknown Up_4_A specific receptor) should also be investigated in physiological and pathophysiological states. A comprehension of the vascular effects of Up_4_A in other cardiovascular diseases, such as atherosclerosis, diabetes, and stroke should also be encouraged.

A better understanding of the role of Up_4_A on vascular function and the regulation of Up_4_A signaling may provide new insights into the mechanisms responsible for cardiovascular diseases and ultimately lead to novel therapeutic strategies with the potential to improve of prognosis of cardiovascular diseases.

## Figures and Tables

**Table 1:** Up_4_A and vascular reactivity.

Artery/tissue	Animal	Response	Putative receptor	Signaling	Reference
Thoracic aorta	Rat	Contraction Relaxation	P1, P2X	L-type Ca^2+^ channel Rho kinase Superoxide NO (endothelium-dependent)	[31]

Pulmonary artery	Rat	Contraction	P2Y	Ca^2+^ (extracellular and intracellular stores)	[36]

Thoracic aorta	Mouse	Contraction Relaxation			[41]

Perfused kidney	Rat	Contraction Relaxation	P2X_1_, P2Y_2_, P2Y_4_ P2Y_1_, P2Y_2_	NO	[14] [37] [37]

Perfused afferent arterioles	Mouse	Contraction			[38]

NO: nitric oxide. Details are shown in the text.
